# The Effects of Cumulative Dose and Polymorphisms in CYP2B6 on the Mitotane Plasma Trough Concentrations in Chinese Patients With Advanced Adrenocortical Carcinoma

**DOI:** 10.3389/fonc.2022.919027

**Published:** 2022-06-30

**Authors:** Xin Liu, Junmei Shang, Qiang Fu, Lin Lu, Jianhua Deng, Yan Tang, Jiantao Li, Dan Mei, Bo Zhang, Shuyang Zhang

**Affiliations:** ^1^ Department of Pharmacy, Peking Union Medical College Hospital, Chinese Academy of Medical Sciences and Peking Union Medical College, Beijing, China; ^2^ State Key Laboratory of Complex Severe and Rare Diseases, Peking Union Medical College Hospital, Beijing, China; ^3^ Chinese Academy of Medical Sciences and Peking Union Medical College, Institute of Materia Medica, Beijing, China; ^4^ Department of Endocrinology, Peking Union Medical College Hospital, Chinese Academy of Medical Sciences and Peking Union Medical College, Beijing, China; ^5^ Department of Urology, Peking Union Medical College Hospital, Chinese Academy of Medical Sciences and Peking Union Medical College, Beijing, China; ^6^ Department of Cardiology, Peking Union Medical College Hospital, Chinese Academy of Medical Sciences and Peking Union Medical College, Beijing, China

**Keywords:** mitotane, pharmacogenetics, cytochrome P450, pregnane X receptor, cumulative dose, therapeutic drug monitoring

## Abstract

Mitotane is the only drug approved to treat adrenocortical carcinoma (ACC), and a relationship of pharmacokinetic/pharmacodynamic has been characterized. However, limited evidence concerning affecting factors in large interindividual variability of the pharmacokinetics of mitotane is available. To address this question, a retrospective analysis was performed on ACC Chinese patients treated with mitotane for more than 3 months. Mitotane plasma trough concentrations were detected at the steady state, and CYP2B6, CYP3A4, and pregnane X receptor (PXR) polymorphisms were genotyped. After examining homogeneous pharmacologic data, we restricted the analyses to 36 patients that received mitotane for a median (interquartile range, IQR) of 9 months (5.00–22.50) with a median dose of 2 g/day (2.00–2.50). As a result, drug exposure was significantly influenced by the cumulative dose of mitotane, and CYP2B6 516GG and CYP2B6 26570CC were at high risk to be below the therapeutic range of mitotane. No association was found between mitotane concentrations with CYP3A4 or PXR polymorphism. Our data firstly indicated that the cumulative dose of mitotane and polymorphisms of CYP2B6 516 and CYP2B6 26570 might significantly affect mitotane plasma trough concentrations in Chinese ACC patients.

## Introduction

Adrenocortical carcinoma (ACC) is a rare aggressive malignancy with an annual incidence of 0.7–2.0 per million people and characterized by a generally poor prognosis (1, 2). The only curative treatment for adrenal cancer in all stages is complete surgical resection (3). Unfortunately, local or metastatic of ACC is frequently recurrent despite tumor being successfully excised (4). For the treatment of ACC, mitotane is the only drug approved by the US Food and Drug Administration and the European Medicine Executive Agency (5). Mitotane acts through the suppression of cell growth and injuring of steroidogenesis in the adrenal glands (6). According to the management guideline of ACC, mitotane is recommended to use for advanced ACC patients with favorable prognostic parameters and the postoperative adjuvant setting following ACC extirpation (7). However, adverse effects related to gastrointestinal tract, neuromuscular, and adrenal insufficiency have been observed in the use of mitotane (7, 8).

A pharmacokinetic/pharmacodynamic relationship of mitotane has been established in patients with ACC (9). Several studies have reported that mitotane plasma concentration ≥14 µg/ml is a predictor of better survival outcome and tumor response, although some of the patients never achieved this level for unknown reasons (7, 10–12). To prevent drug toxicity, the upper limit of the therapeutic range is 20 µg/ml (13). In addition, consensus reports have recently underlined the benefit of reaching and maintaining plasma mitotane trough concentrations in the range of 14–20 µg/ml to maximize efficacy and limit adverse drug events, especially neurological toxicity (7, 14). Meanwhile, the treatment time to reach the target concentrations has been proven to influence the risk of ACC recurrence (15). Thus, therapeutic drug monitoring of mitotane is a crucial clinical strategy to appraise individual treatment response (9, 11). However, it takes 3 months on average to establish a target plasma level of mitotane, and wide individual differences have been observed (16). Several clinical studies have investigated factors involved in the exposure of mitotane between individuals. In particular, the cumulative dose has been shown a higher risk of mitotane accumulation in the Caucasian population, which explains 35% of the variability in the plasma mitotane level (13). Whereas other studies have shown that the level of mitotane has a poor correlation with administration dosage indicates that there are other factors that may affect the target concentrations of mitotane (17, 18). In addition, sex differences in mitotane concentration are still controversial (15, 19), and the unexplained pharmacokinetic variability of mitotane remains high.

Mitotane is principally metabolized into o,p’-DDE and o,p’-DDA in the liver through hydroxylation and oxidation, and it appeared that cytochrome P450 CYP3A4 and CYP2B6 were mostly involved (20). Although CYP3A4 variability in the Caucasian population is limited, the activity of CYP3A enzymes in the Chinese population is highly variable, which contributes to altering the oral bioavailability and systemic clearance of CYP3A substrates (21, 22). To date, however, the influence of *CYP3A4* polymorphisms on mitotane levels has not been reported. Until now, two studies have carried out CYP2B6*6 polymorphisms analysis on mitotane concentrations in Caucasian patients with ACC. One study found that the *GT/TT* genotype of CYP2B6*6 is related to higher mitotane levels (23). The other study also demonstrated that CYP2B6*6 plays a vital role in the changes of mitotane levels for patients with recurrent, not completely resectable, or advanced ACC (24). Moreover, as a transcription factor that adjusts the expression of CYPs, the pregnane X receptor (PXR) may affect mitotane concentration by regulating CYP3A4 and CYP2B6 (25), whereas there is no evidence of the effects of *PXR* polymorphisms on mitotane plasma trough concentrations yet. Thus, in this study, we analyzed the data from a retrospective analysis involving Chinese patients with advanced ACC treated with mitotane for ≥3 months and investigated the effects of genotyping for *CYP2B6*, *3A4*, and *PXR* to identify the factors contributing to the wide interindividual differences in the mean steady-state plasma trough concentration of mitotane.

## Materials and Methods

### Study Design

A retrospective analysis of therapeutic drug monitoring and pharmacogenetic analysis of mitotane was performed from June 2019 to August 2021 in Peking Union Medical College Hospital. The follow-up for this study ended on 1 October 2021. Inclusion criteria for patients of the study were as follows: (1) pathologically diagnosis of ACC; (2) age ≥18 years; (3) detection interval after the last dose of mitotane ≥8 h. Exclusion criteria were as follows: (1) treatment with mitotane for <3 months; (2) incomplete information of mitotane treatment; (3) incomplete follow-up information. A standardized information collection form was applied to review the medical records of each patient. The following information was retrieved for the study: age, sex, weight, height, date of ACC diagnosis, initial date of mitotane treatment, mitotane dose, coadministered drugs, and blood biochemical examination factors. A questionnaire was also sent to patients to collect the data requested for the study. Besides, the data of body mass index (BMI) of ACC patients and cumulative dose of mitotane were further analyzed for the study.

BMI = (weight in kilograms/(height in meters × height in meters))

Cumulative dose of mitotane = daily dose of mitotane × number of days of mitotane treatment prior to plasma sampling.

The protocol of the study was approved by the Ethical Committee of the Peking Union Medical Hospital, Chinese Academy of Medical Sciences (approval number JS-2279) and was conducted following the principles of the Declaration of Helsinki. Written informed consent was obtained from all patients.

### Assessment of Plasma Drug Concentrations

At least 8 h after mitotane administration, 2 ml of blood samples from ACC patients was collected and placed in heparin tubes. For each ACC patient, only one sample was collected. After centrifugation at 3500 r min^−1^ for 10 min, plasma was collected and stored at −60°C until analysis. Mitotane trough plasma levels were analyzed by high-performance liquid chromatographic in our hospital. Briefly, patients’ blood samples were collected in heparin tubes at least 8 h after administration of mitotane, separated the plasma by centrifugation (2,000*g*, 4°C, 10 min), and then stored at −80°C until analysis. After the addition of p,p’-DDD (internal standard), 100 μl of plasma samples containing mitotane was prepared based on simple protein precipitation by adding 50 μl of perchloric acid (5%). The separation was performed on a Shim-pak CLC-ODS (150 × 60 mm, 5 μm) at 35°C using water/acetonitrile (15:85) (pH adjusted to 3.5 by H_3_PO_4_) as mobile phase. The wavelength is 230 nm and the flow rate is 1 ml/min. The calibration curve was linear in the range of 0.5–50 μg/ml.

### Genotyping

Extraction of genomic DNA from peripheral blood samples was carried out using an EasyScript^®^ Quick Extraction Kit (TransGen Biotech, Beijing, China). Samples were genotyped for the most relevant SNPs in these genes: CYP2B6*6 516G > T (rs3745274), *CYP2B6 3003T > C* (rs8100458), *CYP2B6 18492C > T* (rs2279345), *CYP2B6 26570C > T* (rs8192719), *CYP3A4 653G > C* (rs55901263), *CYP3A4 878A > G* (rs28371759), *PXR 24381A > C* (rs1523127), *PXR 7653G > A* (rs6785049), and *PXR 11156A > C* (rs3814057). All genotypes were detected by real-time PCR using TaqMan Assays^®^ (Thermo Fisher Scientific, Waltham, Massachusetts) on ABI 7300 real-time PCR (Thermo Fisher Scientific, Waltham, Massachusetts) according to the manufacturer’s instructions. As quality controls, internal quality controls with known genotypes were used to evaluate genotyping performance in each analysis. All frequencies for the different analysis loci were at the Hardy–Weinberg equilibrium.

### Statistical Analysis

In this study, Kolmogorov–Smirnov test was employed to analyze the continuous variables. Normally distributed variables were expressed as means ± standard deviation (SD) and non-normally distributed variables as median (interquartile range, IQR).

Fisher’s exact tests and Chi-square were used to analyze qualitative data. Mann–Whitney nonparametric test or Student’s t*-*test was applied to compare quantitative data. Spearman’s correlation coefficient was performed to assess the correlation. The Chi-square test was used to estimate genotype frequencies of the various SNPs that deviated from the Hardy–Weinberg equilibrium.

Statistical analysis was performed with SPSS 25.0 software (IBM, Armonk, NY, USA). *P* < 0.05 (two-tailed) was considered statistically significant. Multivariable logistic regression was employed to explore the risk factors for concentrations below 14 μg/ml. The strength of any association was accessed by calculating odds ratios (ORs) and 95% confidence intervals.

## Results

**Figure 1:**
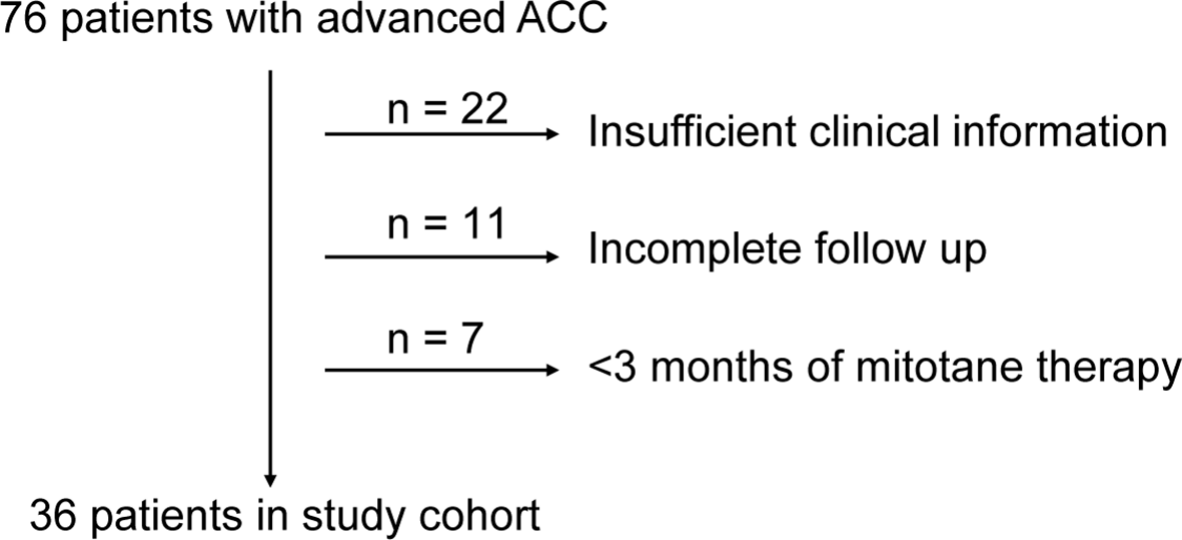
Study cohort.

A total of 76 Chinese ACC patients receiving mitotane were enrolled, 36 patients fulfilled the inclusion/exclusion standards were contained in the study (**Figure 1**). **Table 1** shows the baseline characteristics and genotype frequencies of SNPs of the patients. In this study, the median age of the patients was 47.00 ± 11.00 years and 58% of the patients were females. All patients were between the ages of 27 and 72 years with no differences between sex (46 and 47 years for females and males, respectively, p = 0.42). The median age at ACC diagnosis was 43.00 ± 11.00 years. The mean mitotane dose (± SD) was 2.19 ± 0.60 g/day (1.33 g/m^2^/day), with a median duration of 11.70 months. Thirty patients (83.33%) were coadministrated with hydrocortisone, and more than half (52.78%) were cotreated with CYP enzymes inhibitors.

**Table 1:** Baseline characteristics and genotype frequencies of SNPs of Chinses ACC patients in the present study.

	n (%) or Means ± Standard Deviation
Age, years	47.00 ± 11.00
Sex, no. (%) female	21 (58.33)
Weight, kg	60.00 ± 11.00
Height, cm	165.20 ± 7.40
Body mass index (BMI)^#^, kg/m^2^	21.80 ± 3.60
Age at ACC diagnosis, years	43.00 ± 11.00
cumulative dose^&^, g	561.70 ± 376.00
Detect time, h	11.70 ± 1.10
Mitotane dose, g/day	2.19 ± 0.60
Mitotane dose, g/m^2^	1.33 ± 0.38
Coadministration hydrocortisone	30 (83.33)
Inhibitors of CYP enzymes*, no. (%)	19 (52.78)
Genotyping result	
CYP2B6*6 516 G > T	GG: GT: TT = 18: 16: 2
*CYP2B6 3003 T > C*	TT: TC: CC = 10: 17: 9
*CYP2B6 18492 C > T*	CC: CT: TT = 23: 11: 2
*CYP2B6 26570 C > T*	CC: CT: TT = 20: 12: 4
*CYP3A4 653 G > C*	GG: GC: CC = 26: 7: 3
*CYP3A4 878 A > G*	AA: AG: GG = 26: 10: 0
*PXR 24381 A > C*	AA: AC: CC = 23: 13: 0
*PXR 7653 G > A*	GG: GA: AA = 14: 16: 6
*PXR 11156 A > C*	AA: AC: CC = 11: 13: 12
Blood biochemical examination	
TP, g/L	71.70 ± 5.40
ALB, g/L	41.90 ± 4.60
ALT, U/L	19.10 ± 13.50
AST, U/L	31.80 ± 10.60
TBIL, μmol/L	7.20 ± 2.70
Urea, mmol/L	5.30 ± 1.90
Crea, μmol/L	62.40 ± 16.60
Blood routine examination	
Hemoglobin, g/L	128.80 ± 24.10
Platelets (×10^9^/L)	253.60 ± 92.00
Leukocytes (×10^9^/L)	7.10 ± 6.40
Neutrophils (×10^9^/L)	3.50 ± 1.70
Lymphocytes (×10^9^/L)	1.80 ± 0.90

^#^Body mass index (BMI) is a person’s weight in kilograms divided by the square of height in meters. ^&^Cumulative dose is the total amount of mitotane given to a patient over time. ^*^Inhibitors of CYP included pantoprazole, estazolam, nifedipine, irbesartan and hydrochlorothiazide, omeprazole, indomethacin, and telmisartan.

**Figure 2:**
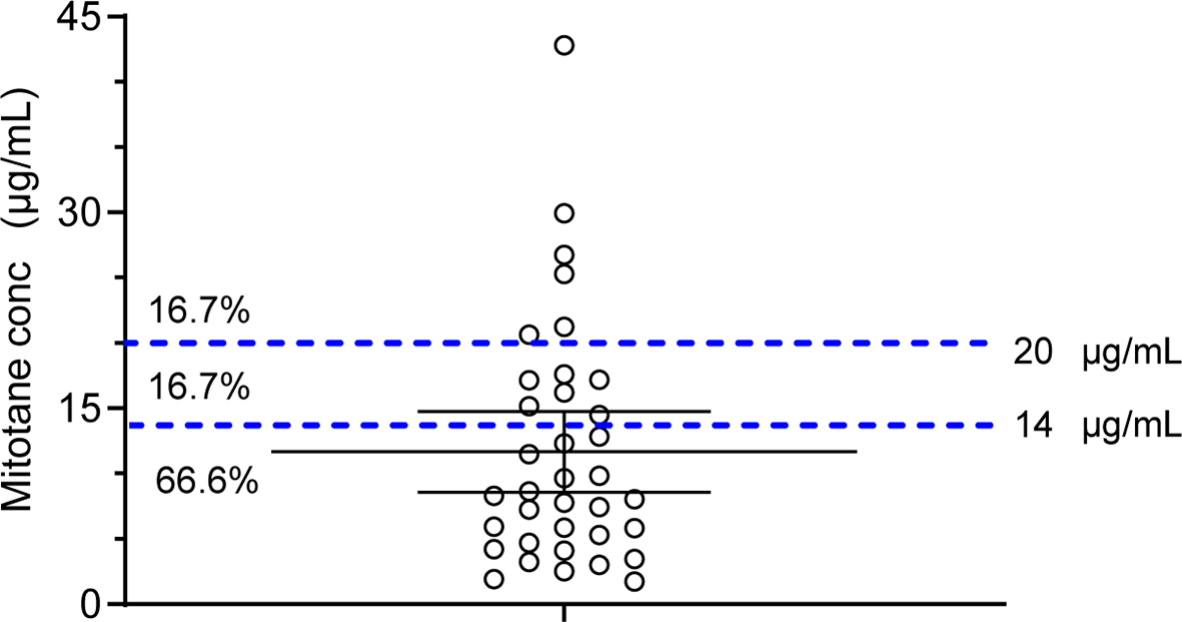
Scatter dot plot of mitotane plasma trough concentrations. Dashed lines represent the concentration therapeutic window. Percentages of samples below, within, or above the therapeutic concentrations are also reported.

A mitotane trough concentration of <14 or >20 μg/ml was defined as outside the therapeutic range. The average plasma trough concentration of mitotane measured in the present study was 8.54 μg/ml (IQR, 4.81–16.93 μg/ml) with no statistically significant differences between sex (males: 10.18 ± 5.83, females: 12.73 ± 10.91 mg/L, p = 0.43). The mitotane trough concentrations in six patients out of 36 (16.70%) were within the therapeutic range, and 66.70 and 16.70% of patients were measured of mitotane trough concentrations below and above the therapeutic range, respectively (**Figure 2**).

In this study, the outcome was a binary variable using the trough concentration of 14 µg/ml. As a result, the median blood mitotane concentration was 5.91 μg/ml (IQR, 3.62–11.10 μg/ml) for patients with the blood mitotane concentration <14 μg/ml (n = 24) and 19.10 μg/ml (IQR, 16.41–26.43 μg/ml) for those with blood mitotane concentration ≥ 14 μg/ml (n=12). The characteristics of both groups are shown in **Table 2**. The mean age, age at diagnosis, height, weight, body mass index, detect time, and the daily dose of mitotane was not shown significant differences for the two groups, whereas the cumulative dose, *CYP2B6 516G > T* and *CYP2B626570 C > T*, was found to be significantly related to plasma trough concentration of mitotane in the univariate analysis. No relationship of the other characteristics as well as laboratory test results and the trough concentration of mitotane was found. Otherwise, mitotane is often combined with hydrocortisone for the treatment of ACC patients. In our study, 19 patients (79.16%) with the blood mitotane concentration <14 μg/ml received hydrocortisone, as compared with 11 patients (91.66%) with blood mitotane concentration >14 μg/ml (*P*=0.34). Furthermore, 13 patients (54.16%) with the blood mitotane concentration <14 μg/ml received inhibitors of CYP enzymes, namely, pantoprazole, estazolam, nifedipine, irbesartan and hydrochlorothiazide, omeprazole, indomethacin, and telmisartan, as compared with six patients (50.00%) with blood mitotane concentration >14 μg/ml (*P* = 0.34). The median blood mitotane concentration was 6.35 μg/ml (IQR, 2.93–13.51 μg/ml) for patients treated with inhibitors of CYP enzymes and 9.20 μg/ml (IQR, 5.70–17.34 μg/ml) for patients who did not receive inhibitors of CYP enzymes (*P* = 0.28). As a result, no significant differences in blood mitotane concentrations for ACC patients when compared with those taking hydrocortisone and inhibitors of CYP enzymes.

**Table 2:** Univariate analysis of factors associated with a low concentration of mitotane (<14 μg/ml).

	Blood Mitotane Concentration		p-value
	< 14 μg/ml (n = 24)	≥ 14 μg/ml (n = 12)	
Age, years	46.00 ± 11.00	48.00 ± 9.00	0.50
Sex, no. (%) female	13 (54.17)	8 (66.67)	0.47
Weight, kg	61.00 ± 12.00	57.00 ± 7.00	0.24
Height, cm	165.70 ± 7.60	164.20 ± 7.20	0.59
Body mass index (BMI), kg/m^2^	22.10 ± 3.90	21.20 ± 2.90	0.46
Age at ACC diagnosis, years	43.00 ± 11.00	45.00 ± 9.00	0.61
cumulative dose, g	388.40 ± 216.30	924.10 ± 387.30	**0.001**
Detect time, h	11.80 ± 0.50	11.50 ± 1.70	0.38
Mitotane dose, g/day	2.23 ± 0.64	2.12 ± 0.53	0.63
Mitotane dose, g/m^2^	1.34 ± 0.40	1.32 ± 0.33	0.89
Coadministration hydrocortisone	19 (79.17)	11 (91.67)	0.34
Inhibitors of CYP enzymes*, no. (%)	13 (54.17)	6 (50.00)	0.81
Genotyping result			
*CYP2B6 516G > T* (TT+GT, %)	9 (37.50)	9 (75.00)	**0.034**
*CYP2B6 3003 T > C* (CC+CT, %)	18 (75.00)	8 (66.67)	0.60
*CYP2B6 18492 C > T* (TT+CT, %)	11 (45.83)	2 (16.67)	0.09
*CYP2B6 26570 C > T* (TT+CT, %)	7 (29.17)	9 (75.00)	**0.002**
*CYP3A4 653 G > C* (CC+GC, %)	5 (20.83)	5 (41.67)	0.19
*CYP3A4 878 A > G* (GG+GA, %)	8 (33.33)	2 (16.67)	0.29
*PXR 24381 A > C* (CC+AC, %)	8 (33.33)	5 (41.67)	0.62
*PXR 7653 G > A* (AA+AG, %)	15 (62.50)	7 (58.33)	0.81
*PXR 11156 A > C* (CC+CA, %)	16 (66.67)	8 (66.67)	1.00
Blood biochemical examination			
TP, g/L	72.30 ± 5.20	70.60 ± 5.90	0.43
ALB, g/L	42.20 ± 5.30	41.20 ± 2.50	0.56
ALT, U/L (≥40)	2 (8.33)	0.00 (0.00)	0.27
AST, U/L (≥35)	8 (33.33)	3 (25.00)	0.61
TBIL, μmol/L	7.50 ± 3.10	6.40 ± 1.70	0.29
Urea, mmol/L	5.50 ± 2.20	4.90 ± 1.10	0.25
Crea, μmol/L	64.50 ± 18.50	57.60 ± 10.40	0.28
Blood routine examination			
Hemoglobin, g/L	129.10 ± 28.60	128.00 ± 9.00	0.90
Platelets (×10^9^/L)	263.30 ± 96.30	232.20 ± 82.20	0.38
Leukocytes (×10^9^/L)	7.80 ± 7.60	5.40 ± 1.70	0.33
Neutrophils (×10^9^/L)	3.70 ± 1.90	3.30 ± 1.50	0.57
Lymphocytes (×10^9^/L)	1.90 ± 1.00	1.70 ± 0.50	0.51

^*^Inhibitors of CYP included pantoprazole, estazolam, nifedipine, irbesartan and hydrochlorothiazide, omeprazole, indomethacin, and telmisartan.

The bold values mean the p-value is < 0.05 and is considered statistically significant.

In the multivariable analysis, the cumulative dose of mitotane, *CYP2B6 516G > T* and *CYP2B6 26570C > T*, was also shown to be significantly and independently related to the trough concentration of mitotane in Chinese ACC patients. Multicollinearity was tested using variables shown in **Table 3**.

**Table 3:** Multivariate logistic regression for potential association with mitotane underexposure (C < 14 mg/L).

Variable	Unadjusted OR (95% CI)	p-value	Adjusted OR (95% CI)	p-value
*CYP2B6 516 G > T*	0.20 (0.043–0.94)	**0.041**	0.15 (0.03-0.49)	**0.034**
*CYP2B6 26570 C > T*	0.14 (0.028–0.66)	**0.013**	0.25 (0.02-0.70)	**0.030**
Cumulative dose	0.94 (0.90–0.91)	**0.004**	0.92 (0.86-0.98)	**0.007**

The bold values mean the p-value is < 0.05 and is considered statistically significant.

**Figure 3:**
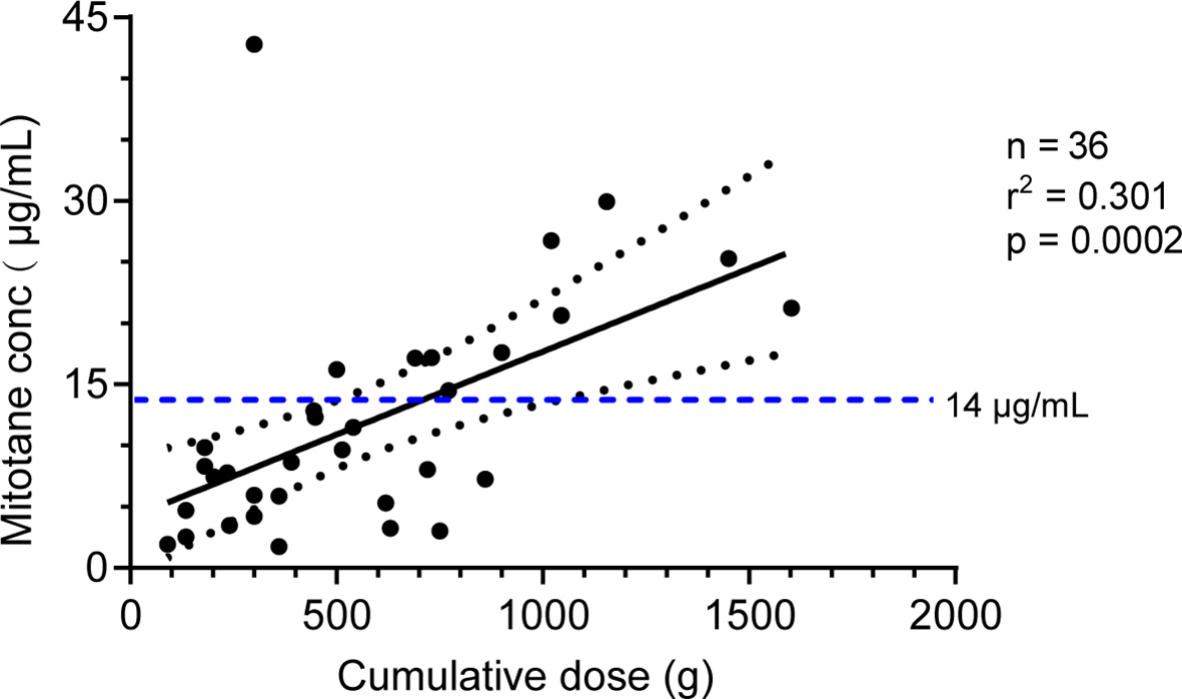
Correlation between the mitotane cumulative dose and the plasma trough level. The solid line represents the linear regression according to y = 0.0137x + 3.968. The dotted lines represent the 95% confidence interval. The horizontal dotted line represents the 14 mg/L therapeutic levels. R: correlation coefficient.

**Figure 4:**
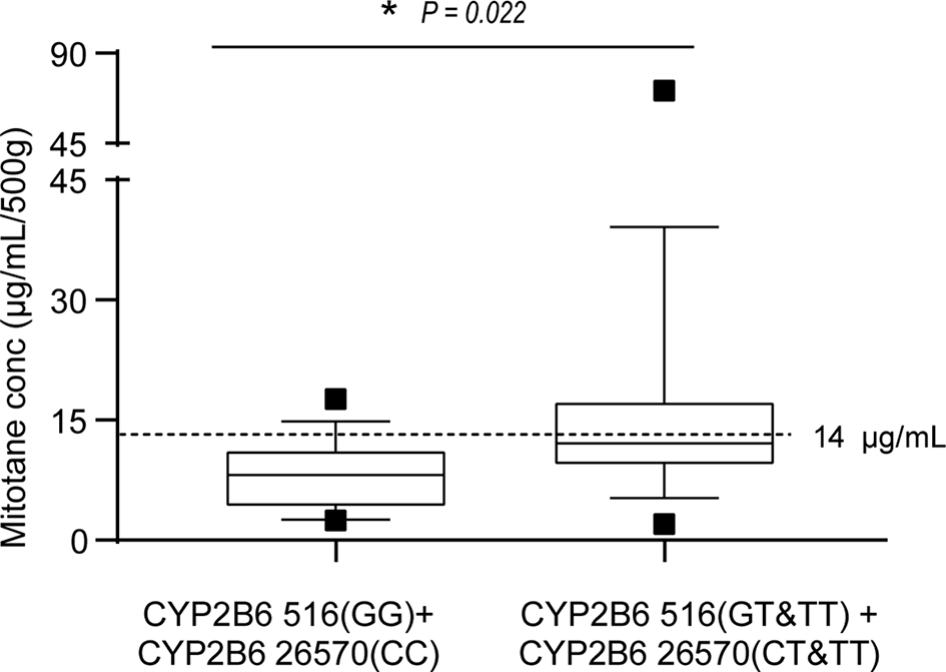
Effects of *CYP2B6 516* and *CYP2B6 26570* polymorphisms on the steady-state mean plasma trough concentrations of mitotane. Graphical analysis was performed using a box and whiskers plot. The box spanned data between two quartiles (IQR), with the median represented as a bold horizontal line. The ends of the whiskers (vertical lines) represent the smallest and largest values that are not outliers. Outliers (black square) are values between 1.5 and 3 IQRs from the end of the box. *P<0.05 vs*. CYP2B6516 (GT&TT)*.

Mitotane trough concentrations were positively associated with cumulative dose as shown in **Figure 3** (r^2^ = 0.301, *P* = 0.0002). We further explored the effect of *CYP2B6516* and *CYP2B626570* polymorphisms on the plasma concentration of cumulative dose-adjusted mitotane. Results shown in patients with both the *CYP2B6516 (GG)* and *CYP2B626570 (CC)* (median 12.10 μg/ml/500g) was about 1.49-fold lower than that in patients with both the *CYP2B6516 (GT&TT)* and *CYP2B626570 (CT&TT)* genotypes (median 8.13 μg/ml/500g; *P* = 0.022; **Figure 4**).

## Discussion

The treatment of ACC is a challenge due to the rarity of the disease. Mitotane has been used to treat advanced ACC since 1960s (26). For patients with ACC, several studies demonstrated that plasma mitotane concentrations ≥14 µg/ml may extend relapse-free survival (16, 27). Since this PK/PD relationship and the narrow therapeutic index of mitotane have been found, interest in the measurement of blood mitotane levels has grown (11, 28–30). However, the factors that affect the variation in the blood mitotane concentrations, especially some of the patients who never achieved this level, still remain unknown. Meanwhile, the information on the real-life use of mitotane for the treatment of patients is limited, making it difficult to guide clinical practice in China. To address those questions, the data related to 36 Chinese ACC patients treated with mitotane have been retrospectively analyzed. The wide interindividual distribution of mitotane concentrations in Chinese patients treated with 2 g/day (IQR, 2.00–2.50 g/day) mitotane has been confirmed. It was also found that the risk of drug underexposure in our population is much greater than overexposure.

After administration, as a lipophilic xenobiotic, mitotane accumulates in adipose tissues and is then released from them. The terminal half-life of mitotane varied from 17 to 159 days (31). Several researchers have conducted PopPK modeling analysis on mitotane in ACC patients (14, 32, 33). One study determined that high-density lipoprotein and triglyceride affect mitotane clearance, whereas the other study found that BMI and self-induced clearance were covariates for the distribution volume of mitotane (32, 33). Another study based on the PK model of mitotane demonstrated the correlations between age, sex, height, weight, and body surface area, and model parameters are weak (34). Nevertheless, no association of weight or BMI with the blood mitotane concentration was found in the present real-world study. Although two studies found that sex can significantly influence the achievement of the therapeutic concentration of mitotane (19, 35), the results of both are controversial. One found that fewer female patients than males were able to achieve and maintain the therapeutic range of 14–20 μg/ml, suggesting that females may be a risk factor for mitotane treatment failure, which presumably stems from effects of sex hormones on drug absorption (19), whereas another study showed that male patients required higher doses of mitotane than female patients to reach the mitotane therapeutic window (35). In the present study, we did not find a relationship between gender and plasma mitotane concentrations. Notably, the studies involved data on female/male hormones, and further prospective studies are needed to confirm the role of gender in the treatment of ACC with mitotane.

In addition, the optimum daily dose of mitotane for ACC treatment is a subject of controversy, and little evidence of the pharmacologic management of mitotane therapy has been published. It was recommended that mitotane treatment starts at a low dose and gradually increases to the maximum tolerated dose, and the daily dose should not exceed 18 g/day to avoid toxicity (36). However, it was found that mitotane daily dose was not related to clinically relevant changes in the blood mitotane level in our study, even if the difference in the blood mitotane concentration was statistically significant, which is consistent with previous results (18). In addition, we found no associations of age, weight, height, age of ACC diagnosis, and treatment time initial date with blood mitotane concentrations and no drug interaction between mitotane and inhibitors of CYP enzymes or antacids. The role of the abovementioned factors might be explained by other factors. According to previous studies, cumulative dose on mitotane concentration is still controversial. The results of a small prospective and multicenter study involving 40 ACC patients, grouped to a low-dose or high-dose mitotane regimen, showed that, despite there being a difference in the average cumulative dose between the two groups, the median maximum plasma concentrations were not significantly different (18). Other literature confirmed a significant correlation of the cumulative dose of mitotane and the highest plasma mitotane trough level (13. In our study, mitotane cumulative dose was significantly associated with trough mitotane concentration in Chinese ACC patients. Most importantly, mitotane cumulative dose is related to mitotane overexposure, whereas factors explaining underexposure of mitotane are currently unclear. To solve this issue, we further investigated the potential involvement of pharmacogenetic factors.

Mitotane has been proved to be a strong and durable inducer of CYP3A4, potentially *via* PXR (31). Arshad et al. assumed that the metabolism of mitotane may be a linear enzyme autoinduction process (37). Our study firstly reports the effects of *CYP2B6*, *CYP3A4*, and *PXR* on mitotane pharmacokinetics in Chinese patients with ACC. Our findings confirmed that mitotane plasma trough concentrations were significantly affected by *CYP2B6 516G > T*, which is consistent with the conception that the mutant “T” allele honors with a lower enzyme activity to metabolize certain drugs (23, 38, 39). In addition, our findings also firstly reported that mitotane levels were significantly influenced by the *CYP2B6 26570C > T* polymorphisms in Chinese patients. The *CYP2B6 26570* “T” allele is also linked to a lower enzyme activity, which caused higher plasma efavirenz concentrations in HIV-infected Thai adults (40, 41). No associations of the *CYP2B6 3003T > C*, *CYP2B6 18492C > T*, *CYP3A4 653G > C*, *CYP3A4 878A > G*, *PXR 24381A > C*, *PXR 7653G > A*, and *PXR 11156A > C* polymorphisms and the mitotane plasma trough concentrations were found in our study. Notably, in patients with both the *CYP2B6 516-GG* and *CYP2B6 26570-CC* genotypes, the cumulative dose-adjusted mitotane concentration in patients was significantly lower than other genotypes.

Our study had several limitations. Firstly, the number of ACC patients who received mitotane therapy included in the study was relatively low, which is related to the fact that mitotane tablets were introduced in China in 2019, while the US Food and Drug Administration has approved mitotane tablets for the treatment of ACC as early as 1970. At present, as the only national-level tertiary hospital in the national collaborative network of hospitals for rare disease diagnosis and treatment, the Peking Union Medical Hospital is the only hospital approved to purchase mitotane, and all ACC patients in the country need to come to our hospital for medical treatment of mitotane. Moreover, because of this limited sample size, the statistical power of the work is also limited and clear conclusions cannot be derived. Therefore, this study is the first description of the use of mitotane in China and is considered hypotheses-generating. Secondly, considering the retrospective study lacks additional patient information, therefore, other possible covariates related to mitotane exposure cannot be excluded. Thirdly, because some polymorphisms were of low frequency, the associations observed may have been accidental. Further prospective clinical evaluation at the initiation of adjuvant mitotane therapy is crucial, which will be able to tailor an individual dose regimen and predict the individual pharmacokinetic responses.

## Conclusions

Our study firstly indicated that the cumulative dose of mitotane and polymorphisms of *CYP2B6 516* and *CYP2B6 26570* might significantly affect mitotane plasma trough concentrations in Chinese ACC patients, which suggests the importance of further prospective clinical investigations.

## Data Availability Statement

The original contributions presented in the study are included in the article/supplementary material. Further inquiries can be directed to the corresponding authors.

## Ethics Statement

The studies involving human participants were reviewed and approved by Chinese Academy of Medical Sciences (approval number JS-2279) was conducted following the principles of the Declaration of Helsinki. Written informed consent was obtained from all patients. The patients/participants provided their written informed consent to participate in this study. Written informed consent was obtained from the individual(s) for the publication of any potentially identifiable images or data included in this article.

## Author Contributions

XL, JS, BZ, and SZ contributed to the conceptualization and design of the study. JS performed the statistical analysis and wrote the original draft. QF, YT, J-TL, LL, JD, and DM assisted with the experiments and contributed to data curation. BZ and SZ administrated the project. All authors contributed to manuscript revision, read, and approved the submitted version.

## Funding

This work was supported by the CAMS Innovation Fund for Medical Sciences (CIFMS 2021-I2M-1-003), the National Key R&D Program of China (2020YFC2008302), the Fundamental Research Funds for the Central Universities (3332021003, 2021-RW310-001), the Youth Research Fund of Peking Union Medical College Hospital (201911755) and the Research Fund of Chinese Research Hospital Association (Y2021FH-YWPJ01-106).

## Conflict of Interest

The authors declare that the research was conducted in the absence of any commercial or financial relationships that could be construed as a potential conflict of interest.

## Publisher’s Note

All claims expressed in this article are solely those of the authors and do not necessarily represent those of their affiliated organizations, or those of the publisher, the editors and the reviewers. Any product that may be evaluated in this article, or claim that may be made by its manufacturer, is not guaranteed or endorsed by the publisher.
